# Consumer Perceptions of Glycation‐Induced Facial Skin Aging and the Role of Skincare Products

**DOI:** 10.1111/jocd.71203

**Published:** 2026-09-18

**Authors:** Sharon Shi, Kelly Chua, Bee Leng Lua

**Affiliations:** ^1^ Groupe Clarins Singapore

**Keywords:** consumer perception, facial skin, glycation, skin aging, skincare

## Abstract

**Background:**

Glycation is a biological process known to exacerbate undesirable skin aging parameters such as yellowing, dullness, wrinkles and reduced elasticity. This study aimed to better understand consumer perceptions of glycation on facial skin and consumer expectations of anti‐glycation skincare.

**Methods:**

Focus group discussions (FGDs) with 24 female participants and an online survey with 800 female participants were conducted in China. Participants were 20–50 years old. The FGDs and survey aimed to determine facial skin qualities consumers associated with glycation and to link these qualities to commonly perceived skincare efficacies. Total unduplicated reach and frequency (TURF) and principal component analyses (PCA) were performed on data collected from the online surveys.

**Results:**

The main expected efficacies of anti‐glycation skincare were reduced dullness and yellowness, and increased radiance and brightness. These efficacies were associated with the skincare descriptive categories “nourishing”, “repairing” and “skin and spot lightening”. TURF analysis showed that the descriptive category “nourishing” had an estimated 50% consumer reach, highlighting that this skincare feature was highly valued in the management of glycation. The descriptive category with the second highest consumer reach was “skin and spot lightening”, however, among women who identified glycation as a skin concern, “repairing” had a greater estimated reach than “skin and spot lightening”.

**Conclusion:**

Female Chinese skincare users mainly associate the descriptive categories “nourishing”, “repairing”, and “skin and spot lightening” with anti‐glycation effects. To combat glycation, women expect to see reduced skin dullness and yellowness and improved skin radiance and brightness.

## 1 Introduction

The biological process of glycation is defined as the modification of proteins by sugars. During the glycation process, monosaccharides, such as glucose, non‐enzymatically modify N‐terminal amino acid groups, arginine residues, and lysine residues of proteins [1]. The process of glycation leads to the formation of advanced glycation end products (AGEs), which have been implicated in the development of several diseases such as diabetes, coronary artery disease, and neurodegenerative disease [1]. AGEs naturally accumulate in the body as we age; however, accumulation can be amplified and exacerbated by exogenous factors such as ultraviolet radiation exposure and a high sugar diet [2].

AGEs accumulate in the skin with age, and have been implicated in skin aging [2]. AGEs alter the composition and structure of skin [3], leading to altered skin pigmentation (yellowing and browning), dullness, deeper wrinkles, and reduced elasticity [3, 4, 5], all of which are clinical signs of skin aging. One mechanism by which AGEs impact skin appearance is through the alteration of collagen structure and turnover [6]. Additionally, AGEs can impair the skin's natural barrier function and slow down repair pathways, making the skin more susceptible to external damage and delayed wound healing [3, 7]. Previous research has found a positive correlation between AGEs and transepidermal water loss (TEWL), and a negative correlation with skin hydration [8, 9]. TEWL, as a key measurement of skin barrier function, assesses stratum corneum performance [9]. This implies AGEs may damage the skin barrier by disrupting its protective capacity and consequently reducing skin hydration. AGEs formation and accumulation in skin have also been shown to play a role in altering skin morphology, leading to increased yellowness and dullness in skin [10, 11].

To counter and prevent glycation and accelerated skin aging, several strategies have been developed. Modulation of key mechanistic pathways has been investigated as a potential anti‐glycation method, including the direct prevention of AGEs formation, inhibition of AGEs signaling through inhibition of the receptor for AGEs (RAGE), and oxidative stress modulation [11, 12, 13]. Several naturally derived compounds, such as polyphenols, polysaccharides, terpenoids, vitamins, alkaloids, and peptides, have been identified as anti‐glycation compounds [14]. In vitro studies also found the cosmetic compounds niacinamide, white water lily extract, lactobionic acid, and artichoke leaf to possess anti‐glycation effects [11].

While the glycating mechanism, glycation‐driven skin aging, and anti‐glycation strategies continue to be key research areas, it remains unclear how consumers recognize, perceive, describe, and link glycation to skin aging. To address this research gap, this study employed qualitative focus group discussions and an online consumer survey to elucidate the skin qualities that Chinese skincare consumers associate with glycation‐induced facial skin aging. Additionally, this study also investigated consumer‐perceived solutions to glycation‐induced facial skin aging.

## 2 Methods

### 2.1 Ethics and Participant Consent

This was a study conducted in female volunteers in China. No ethics approval was sought since the study is exempt from institutional ethical review under Article 32 of the Measures for Ethical Review of Research Involving Human Life Sciences and Medicine (2023) (Document No. 4 [2023] of National Health Commission of China). This research only collected de‐identified subjective skincare perceptions without physical intervention, biological samples, or sensitive personal identifiers; hence, no ethical waiver or official approval was required by national regulations.

All participants provided informed consent prior to participating in the study. During the study, no personally identifiable information was collected. All raw data were anonymized and stored with access restricted to the research team only, and only aggregated statistical results are reported in this manuscript.

### 2.2 Qualitative Focus Group Discussions

Four focus groups (*n* = 6 participants each) were conducted in Shanghai with the aim of understanding consumer perceptions of glycation‐induced skin aging effects and the perceived solutions. Focus groups were conducted by KANTAR consumer research agency between 30 and 31 July 2024. Twenty‐four participants were selected and evenly divided into four groups based on different age brackets and their routine skincare brands (luxury versus premium skincare brands): (1) 25–34‐year‐olds who use luxury skincare brands, (2) 35–50‐year‐olds who use luxury skincare brands, (3) 25–34‐year‐olds who use premium skincare brands, and (4) 35–50‐year‐olds who use premium skincare brands. The main inclusion criteria for the focus discussion groups included the following: female; aged 25–50 years old; college or higher education level; have resided in Shanghai for at least 3 years; any skin type, including sensitive skin; uses ≥ 4 skincare product types (toner, serum, cream, emulsion, eye care, etc.) ≥ 5 times per week; uses ≥ 4 makeup product categories (foundation, eye shadow, contour, etc.) ≥ 3 times per week. Participants were further selected if they had demonstrated awareness, understanding, and concern of skin glycation. All participants expressed awareness and understanding of skin glycation, with 22 of them indicating glycation‐induced skin aging concerns. During the focus group discussions, participants were asked to describe perceived solutions to five common skin conditions: skin puffiness, oxidation, glycation, altered skin metabolism, and reduced turnover. This study only presents the findings on glycation. The findings on oxidation were previously published [15]; the oxidation study and this study are from the same research project, which share the same study design, participant cohort, questionnaire structure, and statistical approach but focus on different topics with completely independent datasets, results, and conclusions. Key emerging themes related to skin glycation are summarized in this study.

Findings from the focus group discussions on glycation‐induced facial skin qualities and the associated skincare descriptive categories were used to develop the online questionnaire for further validation.

### 2.3 Online Survey

The online survey was completed by 800 women aged 20–50 years old, living in either Shanghai (*n* = 200), Beijing (*n* = 200), Chengdu (*n* = 200), or Xi'an (*n* = 200). The survey assessed participants' skincare usage and attitudes toward skincare. The survey also assessed which facial skin qualities they associate with glycation‐induced skin aging, and how these qualities were associated with common skincare product efficacies. The survey was completed via a self‐administered online questionnaire between 13 and 26 September 2024. The complete survey is shown in the Supporting Information section.

The main inclusion criteria for participation in the online survey included the following: female; aged 20–50 years old; any skin type, including sensitive skin; uses ≥ 3 categories of luxury and premium skincare ≥ 5 times per week; uses ≥ 3 categories of makeup ≥ 3 times per week; and monthly household income ≥ renminbi (RMB) 5000.

### 2.4 Data Analyses

#### 2.4.1 Total Unduplicated Reach and Frequency (TURF) Analyses

Iterative TURF analysis simulations were performed to evaluate the optimal combination of skincare attributes related to glycation that maximized consumer reach while minimizing overlap. All descriptive categories were extracted from closed‐ended multiple‐choice survey items, with each response coded as a binary dummy variable (1 = selected, 0 = not selected). Six core categories were retained based on biochemical mechanisms as reported in the literature. Starting from the attribute with the highest selection proportion, the optimal solution path was identified by iteratively eliminating low‐impact attribute combinations to maximize the reach of core anti‐glycation benefit perceptions.

#### 2.4.2 Principal Component Analyses (PCA)

PCA analyses were performed to visualize relationships between attributes and to visualize patterns in the online survey data. PCA analyses were performed using ToolBox, an Excel add‐on designed for PCA.

Two multiple‐choice questions collecting consumer‐perceived anti‐glycation skincare category and corresponding expected efficacy served as raw data for PCA. The selection proportion of each expected efficacy under every skincare category was calculated. This matrix served as the input dataset for PCA analysis. The first two retained principal components jointly explained over 70% of the total variance. A two‐dimensional biplot was generated to visualize the correlation and clustering relationships between skincare category and expected efficacy.

### 2.5 Statistical Analyses

Data from online survey were summarized across the different comparison groups, including demographics (age, city tiers, individual income, user type (luxury versus premium)) and skincare needs (anti‐oxidation, repairing, nourishing and etc.). Online survey data were analyzed using Chi‐squared tests (SPSS software) to determine statistically significant differences between groups at the 95% confidence level.

## 3 Results

### 3.1 Qualitative Focus Group Discussions

#### 3.1.1 Perceived Causes and Effects of Glycation‐Induced Skin Aging

**FIGURE 1:**
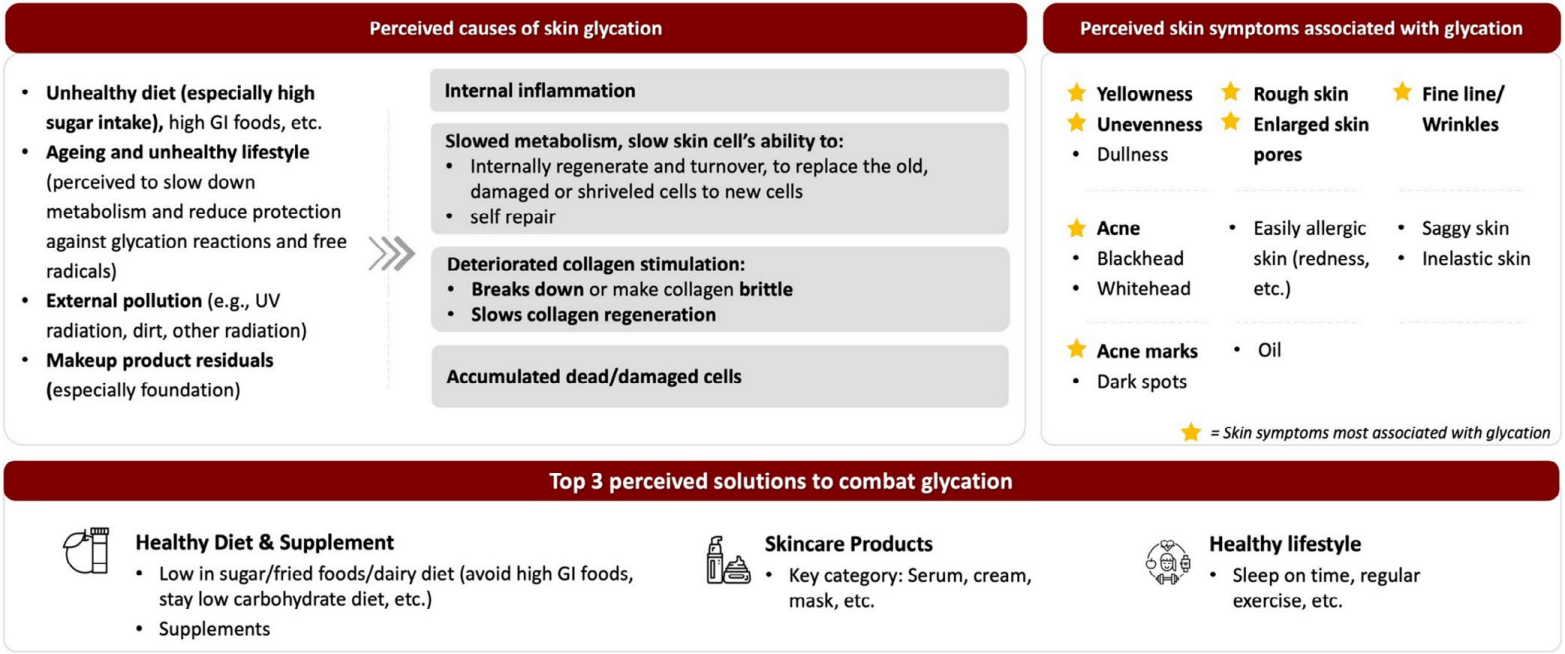
Focus group discussions revealed four perceived causes of glycation, skin symptoms associated with glycation and perceived solutions to combat glycation.

The four main root causes for glycation‐induced skin aging cited by focus group participants were unhealthy diet, aging and unhealthy lifestyle, external pollutants, and makeup residuals (Figure 1), among which high dietary sugar intake was perceived as the key factor. Women perceived these root causes to lead to internal inflammation, slowed skin cell regeneration and turnover, perturbed skin self‐repair, reduced production and increased breakdown of collagen, and accumulation of dead or damaged skin cells (Figure 1).

#### 3.1.2 Perceived Symptoms of Glycation‐Induced Skin Aging

Women identified 15 skin symptoms caused by these biological processes, with the most frequently identified being skin yellowness, unevenness, rough skin, enlarged pores, fine lines and wrinkles, and acne and acne marks (Figure 1).

#### 3.1.3 Perceived Solutions to Combat Glycation‐Induced Skin Aging

Participants considered healthy diet and supplements, skincare products, and a general healthy lifestyle to be the primary solutions to managing the effects of glycation on the skin (Figure 1).

#### 3.1.4 Perceived Effects of Skincare on Glycation‐Induced Skin Aging

Participants were asked how skincare products would help to reduce the visible signs of glycation. Participants most strongly perceived skincare to improve brightness, radiance, even skin tone, and fine and fair skin. These perceived results are associated with the skincare descriptive category “skin and spot lightening”, which was the top ranked category (Table 1). The second highest category identified was “firming”. Other descriptive categories that participants believed could be reversed by skincare products were “repairing” and “anti‐acne” (Table 1).

**TABLE 1:** Perceived effects of skincare on glycation and the descriptive categories associated with these perceived effects.

Perceived effects of skincare on glycation	Descriptive category associated with these perceived effects
Brightness Radiance Even skin tone Fine and fair skin	Skin and spot lightening^a^
Firmness Smoothness with no lines	Firming^a^
Fine and fair skin Tight pores Hydration Acne mark correction Healthy skin (not easily sensitive) Reduced redness	Repairing
Reduced acne	Anti‐acne

^a^ Top associated descriptive categories associated with skin glycation.

It was also cited by some participants that “anti‐glycation” could be another potential descriptive category to address glycation‐induced skin aging effects. They mentioned that skincare products targeting the prevention of AGEs formation can combat glycation‐induced skin aging.

### 3.2 Online Survey

Participants took on average 25 min to complete the survey. The skincare needs of survey respondents have been previously published [15]. In summary, the skincare needs of survey respondents were found to be focused on hydration (selected by 51% of participants) and anti‐wrinkle (selected by approximately 41% of participants).

#### 3.2.1 Perceived Association Between Expected Efficacies and Reduced Glycation‐Induced Skin Aging

**FIGURE 2:**
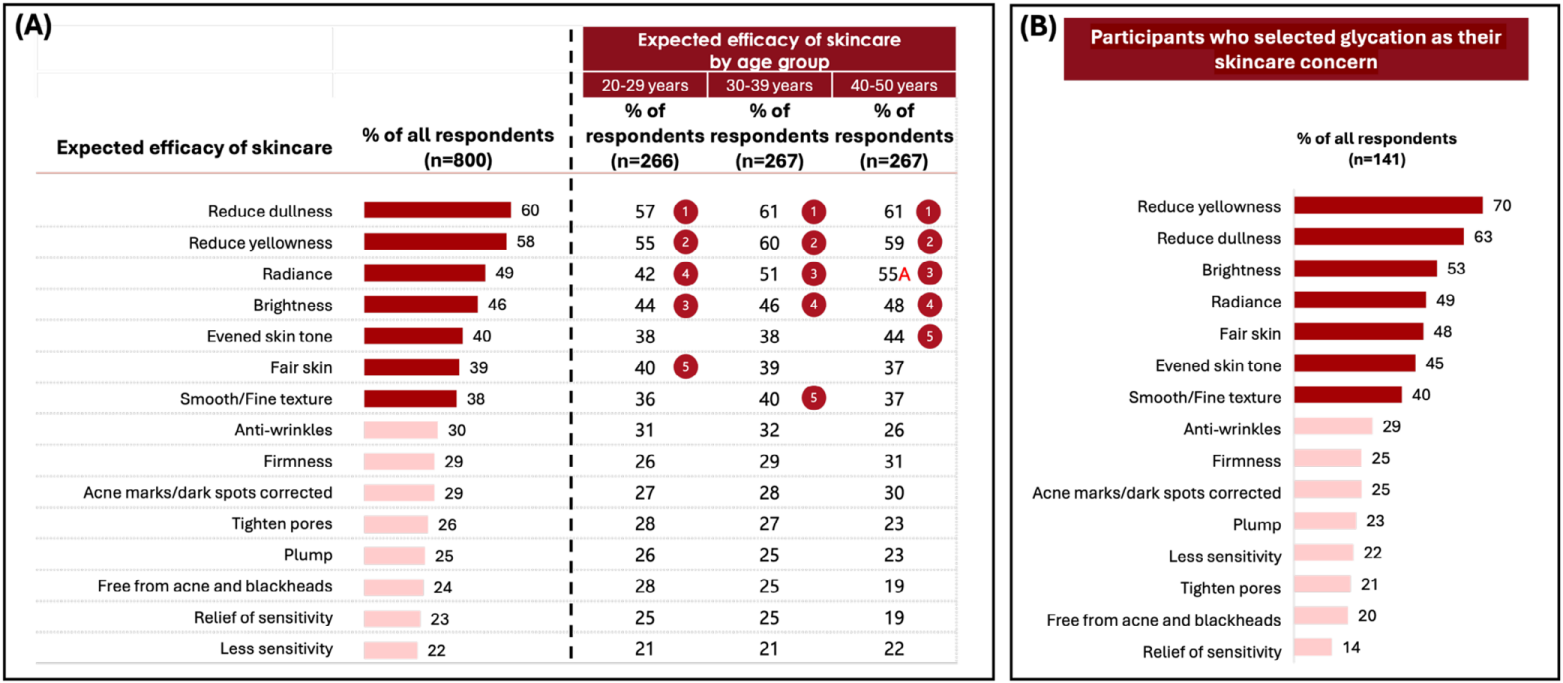
Expected efficacy of skincare for skin glycation (A) by all respondents (*n* = 800) and (B) by respondents who identified glycation as a skin concern (*n* = 141). The numbers shown within a red circle denote the top five skincare needs within that age group. ^A^Significantly higher than the 20–29 years age group as determined by Chi‐squared analysis (*p* ≤ 0.05).

Survey respondents were asked to select skin efficacies that they believed were associated with reducing glycation. The top efficacies that respondents associated with reducing glycation were mainly skin tone‐related benefits including reduced dullness, reduced yellowness, increased radiance, increased brightness, even skin tone, and fair skin, followed by smooth/fine texture (Figure 2A). Reduced dullness and yellowness were the top two ranked efficacies across all three age groups, being selected by 60% and 58% of respondents, respectively. The only statistically significant difference observed between age groups was with the efficacy radiance; this expected efficacy was selected significantly higher by women aged 40–50 years than those aged 20–29 years.

Similar results were seen when examining responses from the subgroup of women who selected glycation as a skin concern (Figure 2B). Reduced yellowness and dullness remained the top two efficacies identified; however, reduced yellowness (selected by 70% of respondents) ranked higher than reduced dullness (63%) in this subgroup.

#### 3.2.2 Perceived Association Between Skincare Descriptive Categories and Reduced Glycation‐Induced Skin Aging

**FIGURE 3:**
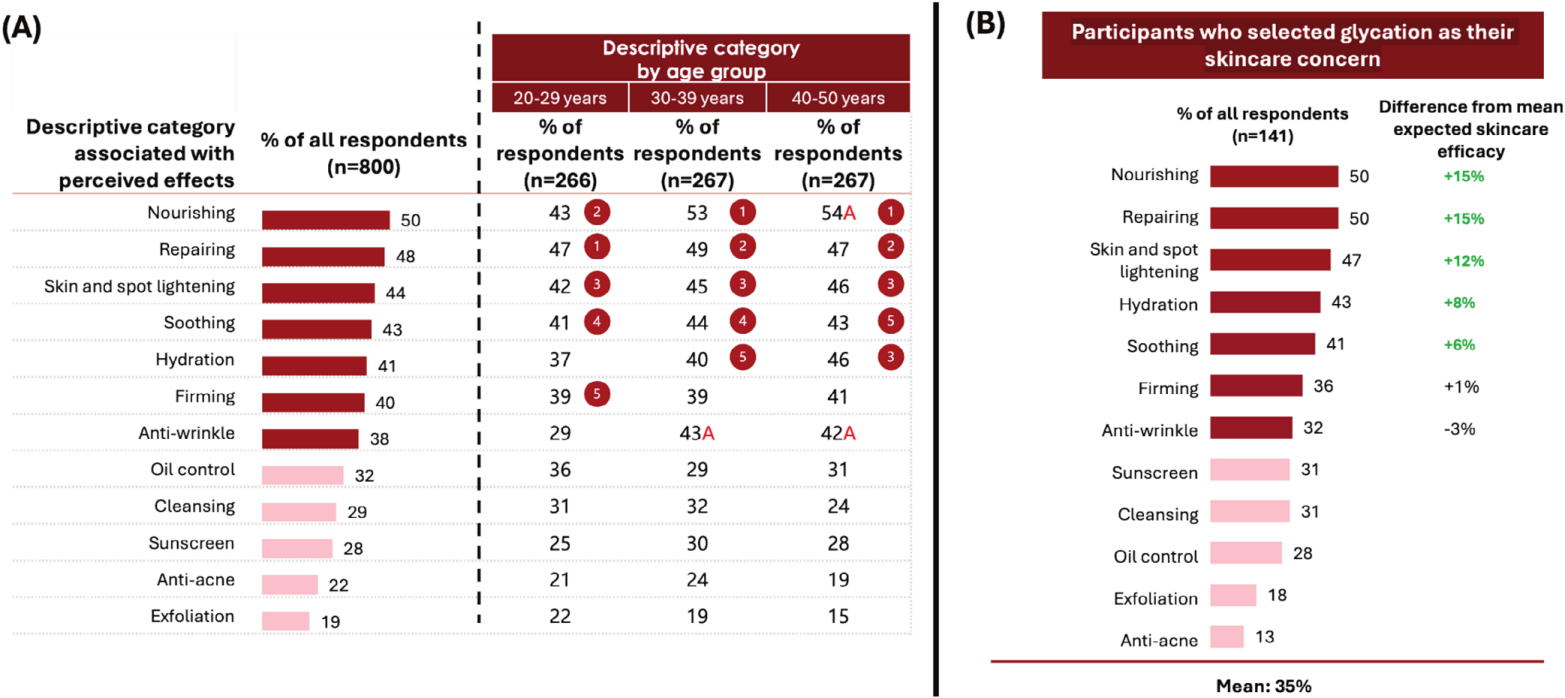
Skincare descriptive categories that were associated with reduced glycation (A) by all respondents (*n* = 800) and (B) by respondents who identified glycation as a skin concern (*n* = 141). The numbers shown within a red circle denote the top five skincare needs within that age group. ^A^Significantly higher than the 20–29 years age group as determined by Chi‐squared analysis (*p* ≤ 0.05).

Survey respondents were asked to select skincare descriptive categories that they believed were associated with reducing glycation. The top five descriptive categories selected by respondents were “nourishing”, “repairing”, “skin and spot lightening”, “soothing”, and “hydration” (Figure 3A). These findings were consistent in the subgroup of women who identified glycation as a skin concern (Figure 3B).

The descriptive categories “nourishing” and “anti‐wrinkle” were ranked significantly higher by women aged 30–39 years and 40–50 years than those aged 20–29 years.

#### 3.2.3 Skincare Product Descriptive Categories and Consumer Reach

**FIGURE 4:**
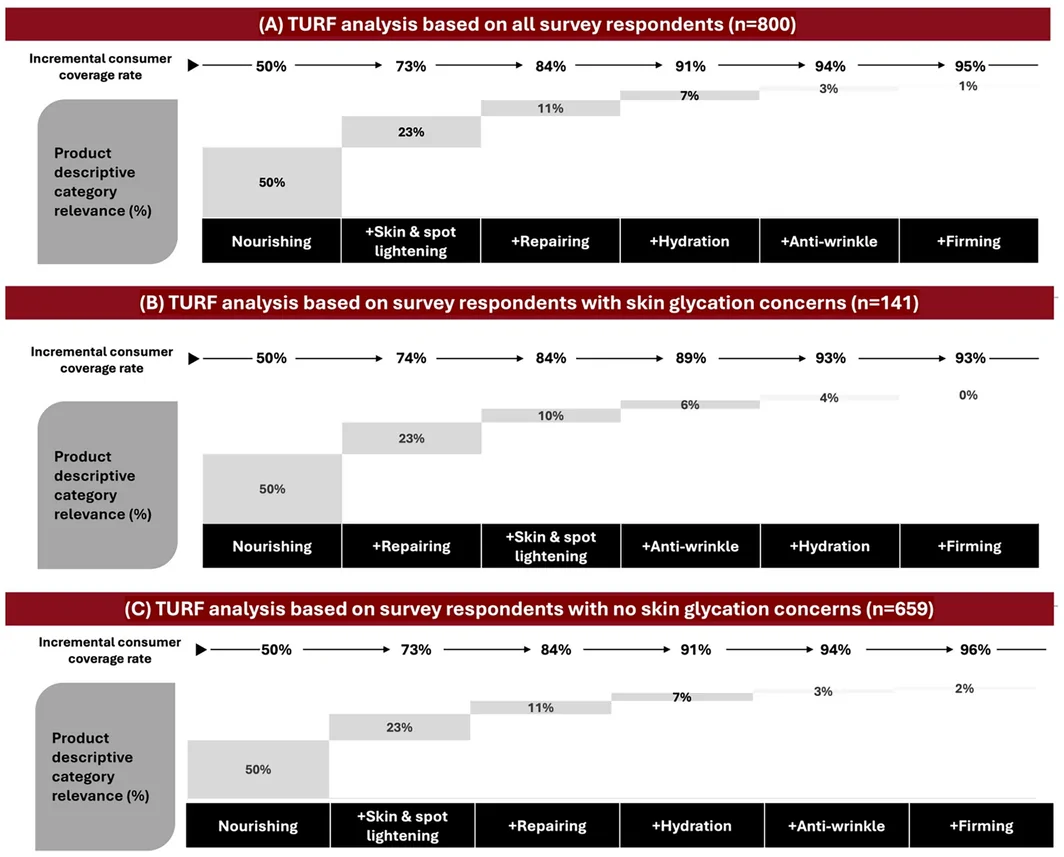
TURF analysis shows the combination of six skincare product descriptive categories that have the highest consumer reach for glycation. (A) shows TURF analysis results based on data from all survey respondents (*n* = 800), (B) shows results based on respondents who indicated skin glycation concerns (*n* = 141), and (C) shows results based on respondents who did not indicate skin glycation concerns (*n* = 659). TURF, total unduplicated reach and frequency.

As reported in literature, glycation on skin can lead to the formation of AGEs, resulting in altered skin pigmentation (yellowing and browning), dullness, deeper wrinkles, reduced elasticity, and impaired skin barrier [2, 3, 4, 7] which in turn can lead to water loss [8]. These skin features were associated with the six descriptive categories “nourishing”, “skin and spot lightening”, “repairing”, “hydration”, “anti‐wrinkle” and “firming” based on the expected efficacies under each category. In the total survey population, TURF analysis using a combination of these six descriptive categories estimated a consumer reach of 95% (Figure 4A). The category “nourishing” alone had a consumer reach of 50%, followed by “skin and spot lightening” (additional 23% consumer reach) and “repairing” (additional 11% consumer reach).

Among respondents who selected glycation as a skin concern (*n* = 141) (Figure 4B), a different pattern was observed whereby “repairing” was more likely to achieve greater additional consumer reach (23%) than “skin and spot lightening” (10%).

Among respondents who did not select glycation as a skin concern (*n* = 659) (Figure 4C), the skincare descriptive category relevance was almost identical to that of the total survey population (Figure 4A).

#### 3.2.4 Principal Component Analysis (PCA) Analysis

**FIGURE 5:**
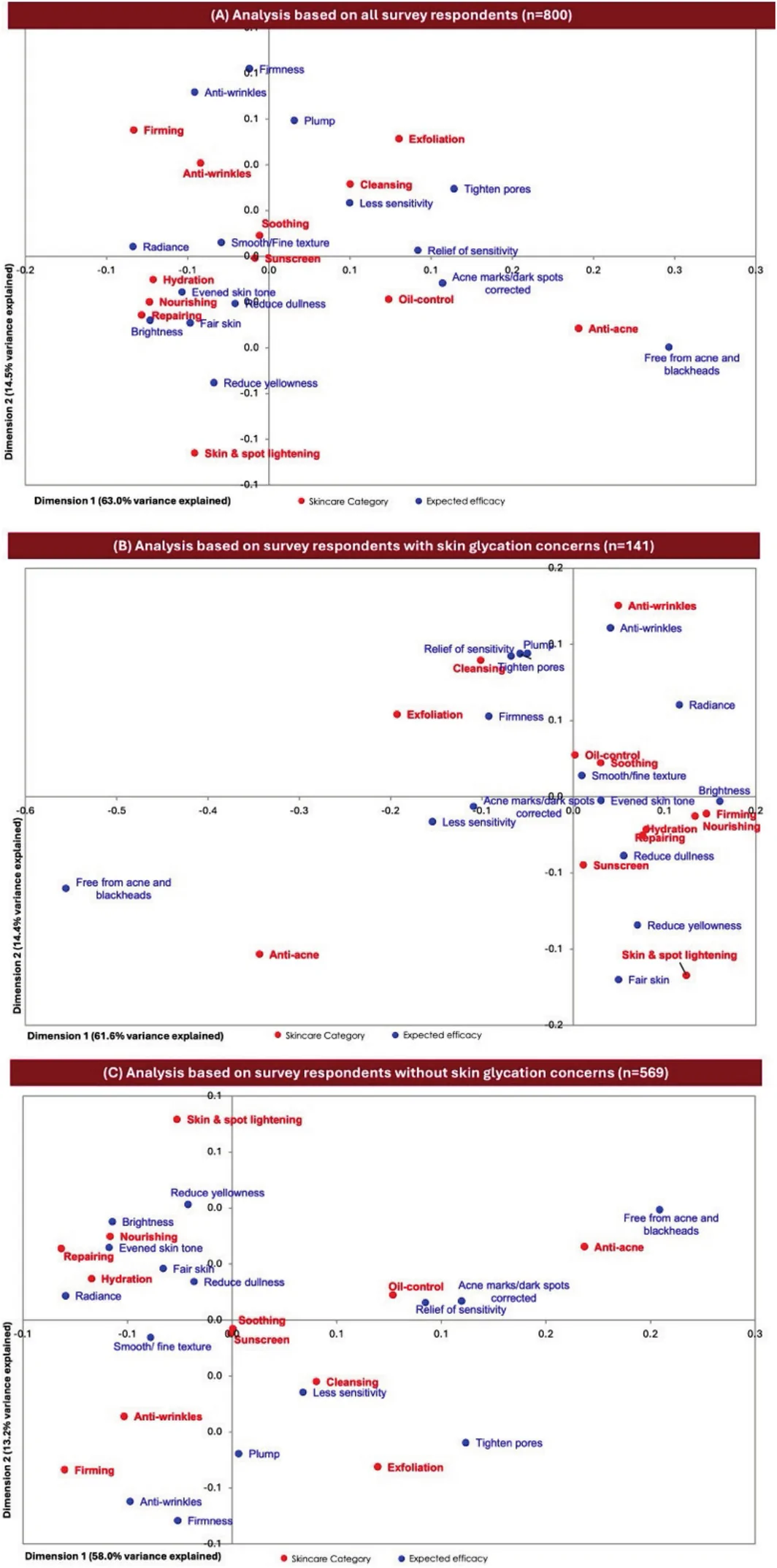
Principal component analysis (PCA) of the online survey data show the level of association of descriptive categories (red) and expected efficacies (blue) based on data from all survey respondents (A), respondents who expressed glycation concerns (B), and respondents who did not express glycation concerns (C). Red dots represent key skincare descriptive categories. The closer the distance between skincare categories, the more similar the expected efficacy they share. Blue dots represent expected efficacy. The closer a blue dot is to a descriptive category (red dot), the more relevant the blue dot is to that category.

Of the 12 skincare descriptive categories analyzed in the total survey population (shown in red; Figure 5A), the descriptive categories “firming”/“anti‐wrinkle”, “skin and spot lightening”, and “anti‐acne” were found to be mutually exclusive.

The descriptive category “skin and spot lightening” was clustered with several skin tone‐related benefits (reduce yellowness, reduced dullness, fair skin, brightness and even skin tone; shown in blue, Figure 5A), suggesting that consumers expect these efficacies under this descriptive category. Other descriptive categories “hydration”, “nourishing”, and “repairing” were perceived less differentiated and clustered with skin tone‐related efficacies (Figure 5A). The smooth/fine texture efficacy was found to be clustered with the descriptive categories “soothing” and “sunscreen”.

Like the total survey population, the descriptive categories “anti‐wrinkle”, “skin and spot lightening”, and “anti‐acne” were mutually exclusive among respondents who selected glycation as a skin concern (Figure 5B). Also like the total survey population, “skin and spot lightening”, together with “hydration”, “nourishing”, and “repairing” were clustered together among respondents who selected glycation as a skin concern, with shared efficacies on skin tone (reduce dullness, even skin tone, and brightness). Interestingly, among respondents who selected glycation as a skin concern, the descriptive categories “sunscreen” and “firming” also clustered with skin tone‐related efficacies.

PCA results from respondents who did not select glycation as a skin concern (Figure 5C) were similar to those seen in the total population (Figure 5A).

#### 3.2.5 Skincare Product Usage and Satisfaction

**FIGURE 6:**
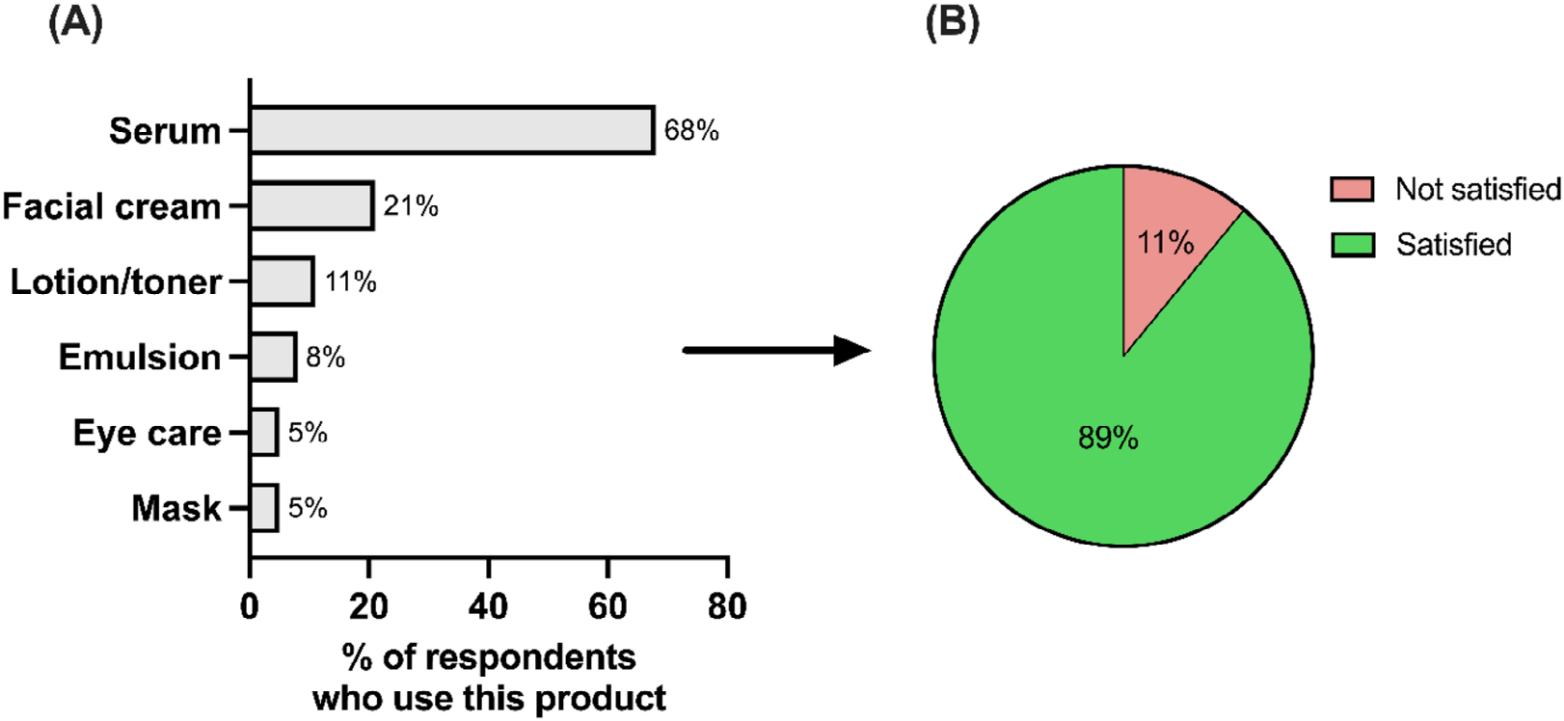
(A) Skincare products that respondents with skin glycation concerns (*n* = 141) use to combat glycation. (B) Respondents' satisfaction rate with these skincare products.

Of the respondents who had skin glycation concerns (*n* = 141), approximately 94% (*n* = 133) of survey respondents used skincare as a solution to glycation. Two‐thirds (68%) of the respondents who had skin glycation concerns used serums; 21% used facial creams; 11% used lotion/toner; 8% used an emulsion; 5% used eye care; and 5% used masks to combat glycation (Figure 6A). The satisfaction rate with the products used was 89% (Figure 6B). The main reason for dissatisfaction in the remaining 11% of participants was a perceived lack of effectiveness.

## 4 Discussion

The objective of this study was to gain a better understanding of the perceptions of female consumers in China toward the impacts of glycation‐induced skin aging, and the efficacies of skincare products in combating glycation‐induced skin aging. Qualitative focus group discussions with small groups of Chinese female consumers with an awareness of glycation; these consumers predominantly associated six skincare efficacies (brightness, radiance, even skin tone, fine and fair skin, firmness, and smooth with no lines) as acting via anti‐glycation mechanisms. These skincare efficacies were associated with the skincare descriptive categories “skin and spot lightening” and “firming”, suggesting that women believe skincare products that reduce glycation will lighten and tighten their skin.

The findings from the online survey supported the focus group discussion results. Chinese female skincare users generally associated reduced glycation with reduced dullness, reduced yellowness, improved radiance, improved brightness, and even skin tone. The main skincare descriptive categories associated with these efficacies were “nourishing”, “repairing”, “skin and spot lightening”, “soothing”, “hydration”, and “firming”. This mostly correlates with the biochemical mechanisms and the impact of glycation on skin aging previously reported in the literature [2, 3, 4, 7, 9]. Interestingly, anti‐wrinkle and firmness were not as highly ranked, implying that women relate the effects of glycation more strongly to visually perceived changes in skin tone rather than skin firmness or wrinkles. Some age‐related differences were observed; the descriptive categories “nourishing” and “anti‐wrinkle” were more frequently associated with glycation by women aged over 30 years, indicating that these women prioritize skin nourishing and anti‐aging measures more than younger women.

In this study, TURF analysis of the survey findings identified an optimal combination of six skincare product descriptive categories (namely “nourishing”, “skin and spot lightening”, “repairing”, “hydration”, “anti‐wrinkle”, and “firming”) that yield the greatest unduplicated consumer reach (95%) for anti‐glycation. Although skin glycation was more reflected in changes in skin tone, the descriptive category “nourishing” had an estimated 50% consumer reach, highlighting that this skincare feature was highly valued in the management of glycation. Women generally associated “nourishing” with skin tone‐related benefits (even skin tone, reduced dullness, fair and brightened skin) based on the PCA results.

The descriptive category with the second highest consumer reach was “skin and spot lightening”. This was found in the total population and among the subpopulation of respondents who did not select glycation as skin concern. However, among respondents who selected glycation as skin concern, the category “repairing” ranked higher than “skin and spot lightening”, indicating that ‘repairing’ is more likely to achieve greater consumer reach for anti‐glycation products.

This study also found that almost all (94%) Chinese skincare consumers used skincare to combat glycation. This demonstrates that skincare consumers perceive skincare products as useful, a finding further supported by an 89% satisfaction rate with anti‐glycation products. The 11% of consumers who expressed dissatisfaction with skincare products for the management of glycation predominantly cited a lack of noticeable effects on their skin. The skincare type most used for glycation is serum, with 68% of women using a serum for anti‐glycation purposes.

The core anti‐glycation‐related descriptive categories “nourishing”, “repairing”, and “skin and spot lightening” identified from the study provide actionable guidance for the cosmetics industry. Brands may prioritize these intuitive perceived benefits in product development and marketing communication, as these categories resonate most with female Chinese skincare users and can effectively enhance consumer recognition and satisfaction. The findings also highlight a notable gap between consumer perceptions and scientific mechanisms of glycation. Consumers mainly associated glycation with visible skin tone changes including yellowness, dullness, and brightness, while largely overlooking the underlying biological impacts such as barrier dysfunction, collagen modification, and elasticity loss. Their perception is likely influenced and shaped by cosmetic marketing claims, social media communication, and beauty industry narratives rather than scientific understanding of AGEs, while this discrepancy reveals prevalent consumer misconceptions and highlights the further need for science‐based consumer education to enhance public awareness of objective skin glycation mechanisms.

Limitations to this study include that the scope of this research was restricted to Chinese females who met a certain income threshold, and were regular users of skincare products (from luxury/premium brands) and makeup products. Meanwhile, focus group participants were prescreened for glycation awareness and understanding. Findings cannot be generalized to all Chinese women and only apply to the highly engaged urban skincare consumers. Future work should expand sampling coverage for wider representativeness.

In conclusion, this study demonstrates that female Chinese skincare users mainly associate the skincare descriptive categories “nourishing”, “repairing”, and “skin and spot lightening” with anti‐glycation effects. In addressing glycation, women expect to see skin tone improvement as the primary focus. Skincare products with the descriptive categories “nourishing”, “repairing”, and “skin and spot lightening” can reach over 80% of the female skincare consumer population. These findings offer insights into how women perceive the effects of glycation on their skin and their expectations from skincare products designed to combat glycation‐induced skin aging effects.

## Author Contributions

B.L.L.: test conception and design; S.S.: data collection and analysis; K.C.: scientific substantiation and efficacy claims.

## Funding

This study was funded by Groupe Clarins, Singapore.

## Ethics Statement

This was a study conducted in female volunteers in China. No ethics approval was sought since the study is exempt from institutional ethical review under Article 32 of the Measures for Ethical Review of Research Involving Human Life Sciences and Medicine (2023) (Document No. 4 [2023] of National Health Commission of China). This research only collected de‐identified subjective skincare perceptions without physical intervention, biological samples or sensitive personal identifiers, hence no ethical waiver or official approval was required by national regulations.

## Consent

All participants provided informed consent prior to participating in the study. During the study, no personally identifiable information was collected. All raw data were anonymized and stored with restricted access for the research team only, and only aggregated statistical results are reported in this manuscript.

## Conflicts of Interest

K.C., B.L.L., and S.S. were employees of Groupe Clarins, Singapore, at the time of the study.

## Supporting information


**Appendix S1:** Skincare claims study: Main questionnaire.

## Data Availability

The data that support the findings of this study are available from the corresponding author upon reasonable request.
