# Supplementary material for: Consumer Perceptions of Glycation‐Induced Facial Skin Aging and the Role of Skincare Products
Source: J Cosmet Dermatol. 2026 Sep 18;25(10):e71203. doi: 10.1111/jocd.71203 (PMC13589218; doi:10.1111/jocd.71203)
Supplement: Supplementary file 1 [file JOCD-25-e71203-s001.docx]

­**Skincare Claims Study**

**Main Questionnaire**

**Skin Conditions**

**K1. Skin type**

| K1 | What is your facial skin type? [Single choice] | Code | Skip |
| --- | --- | --- | --- |
|  | Dry skin | 1 |  |
|  | Combination-dry skin | 2 |  |
|  | Normal skin | 3 |  |
|  | Combination-oily skin | 4 |  |
|  | Oily skin | 5 |  |

**K01. Sensitive-skin status**

| K01 | Do you consider yourself to have sensitive skin? [Single choice] | Code | Skip |
| --- | --- | --- | --- |
|  | Yes | 1 | **K02** |
|  | No | 2 |  |

**K02. Sensitive-skin product usage**

| K02 | **Ask only if K01 = Yes**  When your skin feels sensitive, do you normally use skincare products that are specifically designed for sensitive skin? [Single choice] | Code | Skip |
| --- | --- | --- | --- |
|  | Yes | 1 |  |
|  | No | 2 |  |

**K15. Skincare needs**

| K15 | Which of the following skincare needs do you have? [Multiple choice] | Code | Skip |
| --- | --- | --- | --- |
| (R1) | Skin and spot lightening | 1 |  |
| (R2) | Suncreen | 2 |  |
| (R3) | Anti-acne | 3 |  |
| (R4) | Nourishing | 4 |  |
| (R5) | Repairing | 5 |  |
| (R6) | Anti-wrinkle | 6 |  |
| (R7) | Firming | 7 |  |
| (R8) | Soothing | 8 |  |
| (R9) | Oil-control | 9 |  |
| (R10) | Exfoliation | 10 |  |
| (R11) | Hydration | 11 |  |
| (R12) | Depuffing | 12 | **K16** |
| (R13) | Anti-oxidation | 13 | **K16** |
| (R14) | Anti-glycation | 14 | **K16** |
| (R15) | Skin metabolism | 15 | **K16** |
| (R16) | Anti-dark circle | 16 | **K16** |
|  | Others, please specify: __________ | 98 |  |

**K16. Solutions for selected skincare needs**

| K16 | **Ask only if any of K15 codes 12–16 is selected**  How do you usually address your need for [insert selected items in K15]? [Multiple choice] | Code | Skip |
| --- | --- | --- | --- |
|  | Use skincare products | 1 |  |
|  | Control diet | 2 |  |
|  | Exercise | 3 |  |
|  | Adjust sleep schedule | 4 |  |
|  | Facial massage | 5 |  |
|  | Others, please specify: __________ | 98 |  |

**H2. Hidden Question - Skincare Demand Quota**

| H2 | Skincare demand quota  **Code1-5 minimum 120 readable samples per cell, balanced by city** | | Code | Skip |
| --- | --- | --- | --- | --- |
|  | Depuffing | **K15=12 and K16=1** | 1 |  |
|  | Anti-oxidation | **K15=13 and K16=1** | 2 |  |
|  | Anti-glycation | **K15=14 and K16=1** | 3 |  |
|  | Skin metabolism | **K15=15 and K16=1** | 4 |  |
|  | Anti-dark circle | **K15=16 and K16=1** | 5 |  |

**Main Questionnaire**

**Depuffing**

**DP1. Depuffing: expected effects**

| DP1 | Which of the following effects do you believe can be achieved through depuffing? [Multiple choice] | Code | Skip |
| --- | --- | --- | --- |
| (R1) | Firming & lifting | 1 |  |
| (R2) | V-shape | 2 |  |
| (R3) | Defined jawline | 3 |  |
| (R4) | Defined contour | 4 |  |
| (R5) | Smooth with no lines | 5 |  |
| (R6) | Comfort | 6 |  |
| (R7) | Calming and soothing | 7 |  |
| (R8) | Plump | 8 |  |
| (R9) | High skin turgor | 9 |  |
| (R10) | Elastic skin | 10 |  |
| (R11) | Reduce eye puffiness | 11 |  |
| (R12) | Reduce swelling | 12 |  |
| (R13) | Reduce eye fatigue | 13 |  |
|  | Others, please specify: __________ | 98 |  |

**DP2. Depuffing: category relevance**

| DP2 | Which skincare benefit categories do you associate with depuffing? [Multiple choice] | Code | Skip |
| --- | --- | --- | --- |
|  | Nourishing | 4 |  |
|  | Repairing | 5 |  |
|  | Anti-wrinkle | 6 |  |
|  | Firming | 7 |  |
|  | Soothing | 8 |  |
|  | Others, please specify: __________ | 98 |  |
|  | None of the above | 99 |  |

**DP3. Depuffing: expected time**

| DP3 | How soon do you expect to see depuffing effects after using a skincare product? [Single choice] | Code | Skip |
| --- | --- | --- | --- |
|  | Immediately | 1 |  |
|  | Within half a day | 2 |  |
|  | Within 1 day | 3 |  |
|  | Within 1 week | 5 |  |
|  | More than 1 week | 6 |  |

**DP4-DP6** **ask only if H2=1**

**DP4. Depuffing: product usage**

| DP4 | Which skincare products do you use to address puffiness? Please write at least one brand and product name.  Brand 1: __________ Product name 1: __________  Brand 2: __________ Product name 2: __________  Brand 3: __________ Product name 3: __________ |
| --- | --- |
|  |  |

**DP5. Depuffing: satisfaction**

| DP5 | Overall, how satisfied are you with the skincare products you currently use for puffiness? Rate 1-5, 5=very satisfied,1=very unsatisfied. [Single choice] | Code | Skip |
| --- | --- | --- | --- |
|  | 5 | 5 |  |
|  | 4 | 4 |  |
|  | 3 | 3 |  |
|  | 2 | 2 |  |
|  | 1 | 1 |  |

**DP6. Depuffing: low satisfaction reason**

| DP6 | **Only if DP5=1-3**  What are the reasons for your low satisfaction with the products you use for puffiness? [Open-ended]  _________________________________________________________________________________ |
| --- | --- |

**Oxidation**

**O1. Oxidation: expected effects**

| O11 | Which of the following effects do you believe can be achieved through anti-oxidation within a short term (within one week)? [Multiple choice] | Code | Skip |
| --- | --- | --- | --- |
| (R1) | Brightness | 1 |  |
| (R2) | Reduce dullness | 2 |  |
| (R3) | Radiance | 3 |  |
| (R4) | Evened skin tone | 4 |  |
| (R5) | Smooth/Fine texture | 5 |  |
| (R6) | Relief of sensitivity | 6 |  |
| (R7) | Hydrated | 7 |  |
| (R8) | Oil-water balance | 8 |  |
|  | Others, please specify: __________ | 98 |  |

| O12 | Which of the following effects do you believe can be achieved through long-term (more than one week) anti-oxidation? [Multiple choice] | Code | Skip |
| --- | --- | --- | --- |
| (R1) | Smooth/Fine texture | 1 |  |
| (R2) | Fair skin | 2 |  |
| (R3) | Reduce yellowness | 3 |  |
| (R4) | Firmness | 4 |  |
| (R5) | Plump | 5 |  |
| (R6) | Tighten pores | 6 |  |
| (R7) | Acne marks/dark spots corrected | 7 |  |
| (R8) | Less sensitivity | 8 |  |
| (R9) | Oil-water balance | 9 |  |
| (R10) | Anti-wrinkles | 10 |  |
|  | Others, please specify: __________ | 98 |  |

**O2. Oxidation: category relevance**

| O2 | Which skincare benefit categories do you associate with anti-oxidation? [Multiple choice] | Code | Skip |
| --- | --- | --- | --- |
| (R1) | Skin and spot lightening | 1 |  |
| (R2) | Sunscreen | 2 |  |
| (R3) | Anti-acne | 3 |  |
| (R4) | Nourishing | 4 |  |
| (R5) | Repairing | 5 |  |
| (R6) | Anti-wrinkle | 6 |  |
| (R7) | Firming | 7 |  |
| (R8) | Soothing | 8 |  |
| (R9) | Oil control | 9 |  |
| (R10) | Exfoliation | 10 |  |
| (R11) | Hydration | 11 |  |
| (R12) | Cleansing | 12 |  |
|  | Others, please specify: __________ | 98 |  |
|  | None of the above | 99 |  |

**O3. Oxidation: expected time**

| O31 | How soon do you expect to see the short-term anti-oxidation effects you selected (insert O11 options) after using a skincare product? [Single choice] | Code | Skip |
| --- | --- | --- | --- |
|  | Immediately | 1 |  |
|  | Within half a day | 2 |  |
|  | Within 1 day | 3 |  |
|  | Within 1 week | 5 |  |

| O32 | How soon do you expect to see the long-term anti-oxidation effects you selected (insert O12 options) after using a skincare product? [Single choice] | Code | Skip |
| --- | --- | --- | --- |
|  | Within 2 weeks | 1 |  |
|  | Within 1 month | 2 |  |
|  | 1–3 months | 3 |  |
|  | 3–6 months | 4 |  |
|  | 6–12 months | 5 |  |
|  | More than 1 year | 6 |  |

**O4-O6 ask only if H2 = 2**

**O4. Oxidation: product usage**

| O4 | Which skincare products do you use to address oxidation? Please write at least one brand and product name.  Brand 1: __________ Product name 1: __________  Brand 2: __________ Product name 2: __________  Brand 3: __________ Product name 3: __________ |
| --- | --- |
|  |  |

**O5. Oxidation: satisfaction**

| O5 | Overall, how satisfied are you with the products you use for oxidation? Rate 1–5, 5=very satisfied, 1=very unsatisified. [Single choice] | Code | Skip |
| --- | --- | --- | --- |
|  | 5 | 5 |  |
|  | 4 | 4 |  |
|  | 3 | 3 |  |
|  | 2 | 2 |  |
|  | 1 | 1 |  |

**O6. Oxidation: low satisfaction reason**

| O6 | **Ask only if O5 = 1–3**  What are the reasons for your low satisfaction with the products you use for oxidation? [Open-ended]  _________________________________________________________________________________ |
| --- | --- |

**Skin Metabolism**

**M1. Skin Metabolism: expected effects**

| M1 | Which of the following effects do you believe can be achieved by improving skin metabolism? [Multiple choice] | Code | Skip |
| --- | --- | --- | --- |
| (R1) | Brightness | 1 |  |
| (R2) | Reduce dullness | 2 |  |
| (R3) | Refresh/mattified | 3 |  |
| (R4) | Radiance | 4 |  |
| (R5) | Rosy | 5 |  |
| (R6) | Hydrate | 6 |  |
| (R7) | Smooth/Fine texture | 7 |  |
| (R8) | Skincare products well absorbed | 9 |  |
| (R9) | Smooth/seamless makeup finish | 10 |  |
| (R10) | Depuffing | 11 |  |
| (R11) | Less sensitivity | 12 |  |
| (R12) | Relief of sensitivity | 13 |  |
| (R13) | Fortify skin barrier | 14 |  |
| (R14) | Acne marks/dark spots corrected | 15 |  |
|  | Others, please specify: __________ | 98 |  |

**M2. Skin Metabolism: category relevance**

| M2 | Which skincare benefit categories do you associate with improving skin metabolism? [Multiple choice] | Code | Skip |
| --- | --- | --- | --- |
| (R1) | Skin and spot lightening | 1 |  |
| (R2) | Sunscreen | 2 |  |
| (R3) | Anti-acne | 3 |  |
| (R4) | Nourishing | 4 |  |
| (R5) | Repairing | 5 |  |
| (R6) | Anti-wrinkle | 6 |  |
| (R7) | Firming | 7 |  |
| (R8) | Soothing | 8 |  |
| (R9) | Oil control | 9 |  |
| (R10) | Exfoliation | 10 |  |
| (R11) | Hydration | 11 |  |
| (R12) | Cleansing | 12 |  |
|  | Others, please specify: __________ | 98 |  |
|  | None of the above | 99 |  |

**M3. Skin Metabolism: expected time**

| M3 | How soon do you expect to see effects from improving skin metabolism after using a skincare product? [Single choice] | Code | Skip |
| --- | --- | --- | --- |
|  | Within 1 week | 1 |  |
|  | Within 2 weeks | 2 |  |
|  | Within 1 month | 3 |  |
|  | 1–3 months | 4 |  |
|  | 3–6 months | 5 |  |
|  | 6–12 months | 6 |  |
|  | More than 1 year | 7 |  |

**M4-M6 ask only if H2 = 4**

**M4. Skin Metabolism: product usage**

| M4 | Which skincare products do you use to improve skin metabolism? Please write at least one brand and product name.  Brand 1: __________ Product name 1: __________  Brand 2: __________ Product name 2: __________  Brand 3: __________ Product name 3: __________ |
| --- | --- |
|  |  |

**M5. Skin Metabolism: satisfaction**

| M5 | Overall, how satisfied are you with the products you use to improve skin metabolism? Rate 1–5, 5=very satisfied,1=very unsatisfied. [Single choice] | Code | Skip |
| --- | --- | --- | --- |
|  | 5 | 5 |  |
|  | 4 | 4 |  |
|  | 3 | 3 |  |
|  | 2 | 2 |  |
|  | 1 | 1 |  |

**M6. Skin Metabolism: low satisfaction reason**

| M6 | **Ask only if M5 = 1–3**  What are the reasons for your low satisfaction with the products you use to improve skin metabolism? [Open-ended]  _________________________________________________________________________________ |
| --- | --- |

**Glycation**

**G1. Glycation: expected effects**

| G1 | Which of the following effects do you believe can be achieved through anti-glycation? [Multiple choice] | Code | Skip |
| --- | --- | --- | --- |
| (R1) | Brightness | 1 |  |
| (R2) | Reduce dullness | 2 |  |
| (R3) | Radiance | 3 |  |
| (R4) | Evened skin tone | 4 |  |
| (R5) | Smooth/Fine texture | 5 |  |
| (R6) | Fair skin | 6 |  |
| (R7) | Reduce yellowness | 7 |  |
| (R8) | Firmness | 8 |  |
| (R9) | Anti-wrinkles | 9 |  |
| (R10) | Plump | 10 |  |
| (R11) | Tighten pores | 11 |  |
| (R12) | Acne marks/dark spots corrected | 12 |  |
| (R13) | Free from acne and blackheads | 13 |  |
| (R14) | Less sensitivity | 14 |  |
| (R15) | Relief of sensitivity | 15 |  |
|  | Others, please specify: __________ | 98 |  |

**G2. Glycation: category relevance**

| G2 | Which skincare benefit categories do you associate with anti-glycation? [Multiple choice] | Code | Skip |
| --- | --- | --- | --- |
| (R1) | Skin and spot lightening | 1 |  |
| (R2) | Sunscreen | 2 |  |
| (R3) | Anti-acne | 3 |  |
| (R4) | Nourishing | 4 |  |
| (R5) | Repairing | 5 |  |
| (R6) | Anti-wrinkle | 6 |  |
| (R7) | Firming | 7 |  |
| (R8) | Soothing | 8 |  |
| (R9) | Oil control | 9 |  |
| (R10) | Exfoliation | 10 |  |
| (R11) | Hydration | 11 |  |
| (R12) | Cleansing | 12 |  |
|  | Others, please specify: __________ | 98 |  |
|  | None of the above | 99 |  |

**G3. Glycation: expected time**

| G3 | How soon do you expect to see anti-glycation effects after using a skincare product? [Single choice] | Code | Skip |
| --- | --- | --- | --- |
|  | Within 1 week | 1 |  |
|  | Within 2 weeks | 2 |  |
|  | Within 1 month | 3 |  |
|  | 1–3 months | 4 |  |
|  | 3–6 months | 5 |  |
|  | 6–12 months | 6 |  |
|  | More than 1 year | 7 |  |

**程序员：G4-G6 ask only if H2 = 3**

**G4.** **Glycation: product usage**

| G4 | Which skincare products do you use to address glycation? Please write at least one brand and product name.  Brand 1: __________  Product name 1: __________  Brand 2: __________  Product name 2: __________  Brand 3: __________  Product name 3: __________ |
| --- | --- |
|  |  |

**G5. Glycation: satisfaction**

| G5 | Overall, how satisfied are you with the products you use for glycation? Rate 1–5, 5=very satisfied, 1=very unsatisfied. [Single choice] | Code | Skip |
| --- | --- | --- | --- |
|  | 5 | 5 |  |
|  | 4 | 4 |  |
|  | 3 | 3 |  |
|  | 2 | 2 |  |
|  | 1 | 1 |  |

**G6. Glycation: low satisfaction reason**

| G6 | **Ask only if G5=1/2/3**  What are the reasons for your low satisfaction with the products you use for glycation? [Open-ended]  _________________________________________________________________________________ |
| --- | --- |

**Exfoliation**

**EA1. Exfoliation: expected effects**

| EA1 | | Which of the following effects do you believe can be achieved through exfoliation? [Multiple choice] | | Code | Skip |
| --- | --- | --- | --- | --- | --- |
| (R1) |  | | Tighten pores | 1 |  |
| (R2) |  | | Remove blackheads | 3 |  |
| (R3) |  | | Refresh/mattified | 5 |  |
| (R4) |  | | Oily-free | 6 |  |
| (R5) |  | | Skincare products well absorbed | 7 |  |
| (R6) |  | | Smooth/seamless makeup finish | 8 |  |
| (R7) |  | | Comfort | 9 |  |
| (R8) |  | | Skin breathability | 10 |  |
| (R9) |  | | Reduce roughness | 11 |  |
| (R10) |  | | Smoother skin | 12 |  |
| (R11) |  | | Radiance/brightness | 13 |  |
| (R12) |  | | Reduce dullness | 14 |  |
| (R13) |  | | Skin is renewed/regenerated/Resurface the skin | 15 |  |
| (R14) |  | | Improve/refine skin texture | 16 |  |
|  |  | | Others, please specify: __________ | 98 |  |

**EA2. Exfoliation: product categories**

| EA2 | Which of the following product types do you associate with exfoliation? [Multiple choice] | Code | Skip |
| --- | --- | --- | --- |
|  | Cleansing product | 1 |  |
|  | Lotion/Toner | 2 |  |
|  | Acid serum | 3 |  |
|  | Cleansing mask | 11 |  |
|  | Sheet mask | 12 |  |
|  | Makeup remover | 13 |  |
|  | Facial scrub | 14 |  |
|  | Others, please specify: __________ | 98 |  |
|  | None of the above | 99 |  |

**End of the questionnaire**
